# Supplementary figures and images for: TGFβ signalling is required to maintain pluripotency of human naïve pluripotent stem cells
Source: eLife. 2021 Aug 31;10:e67259. doi: 10.7554/eLife.67259 (PMC8410071; doi:10.7554/eLife.67259)

Figure 1 - Source data 1

a

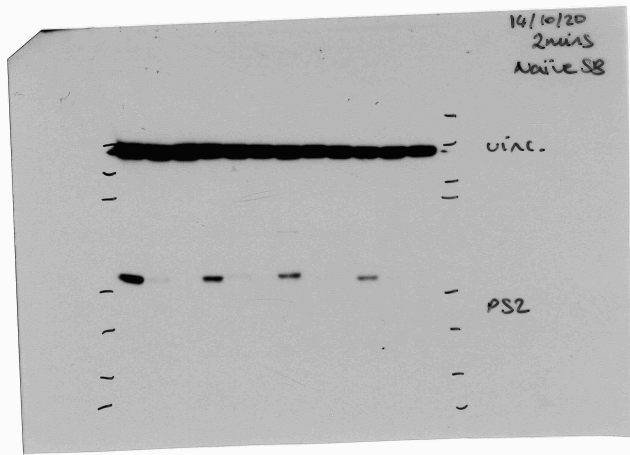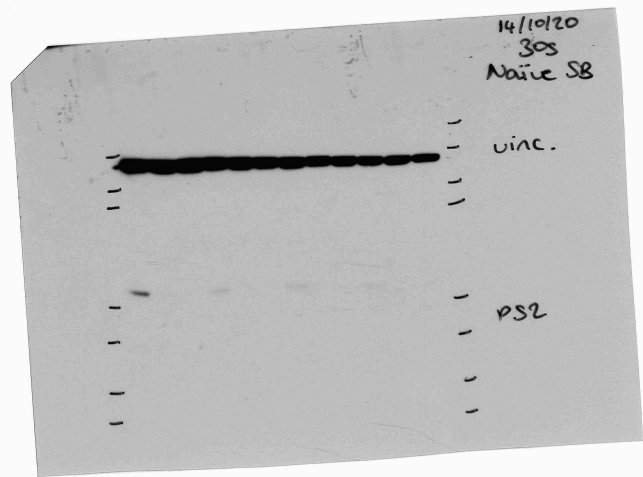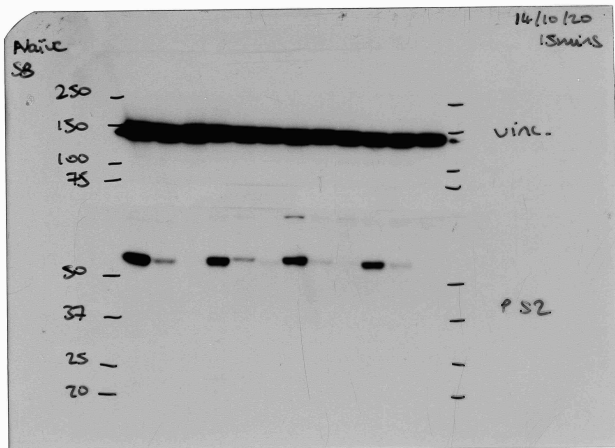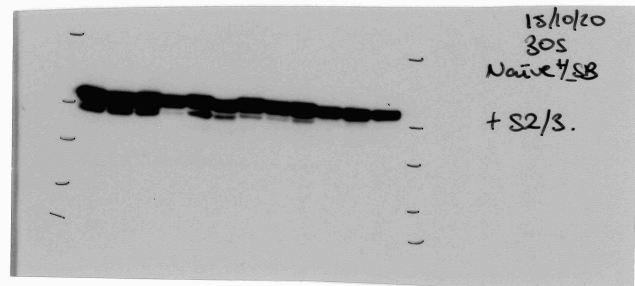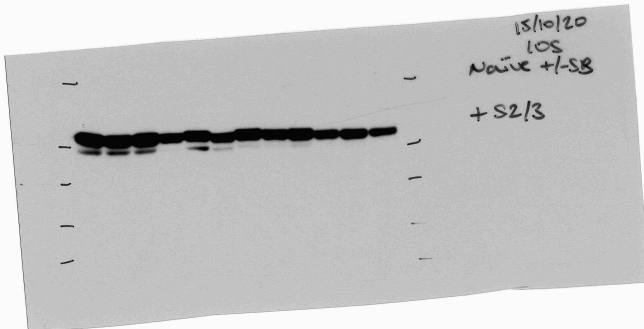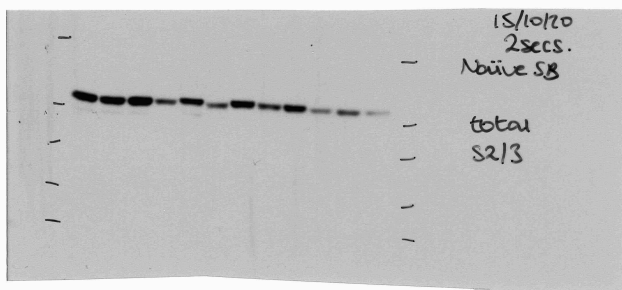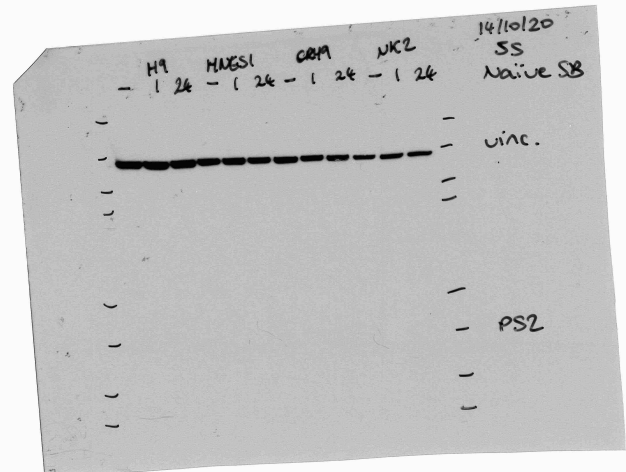

b

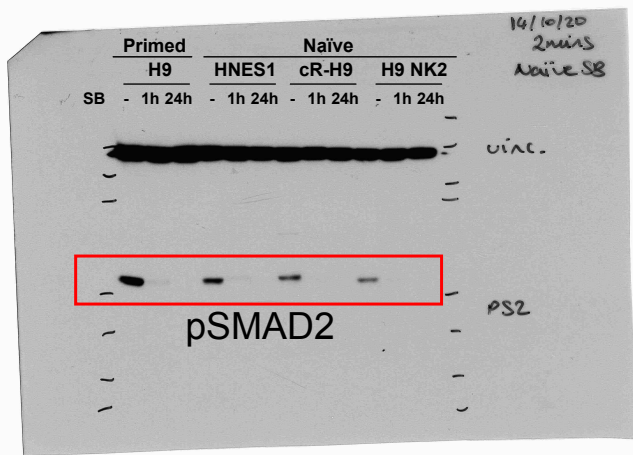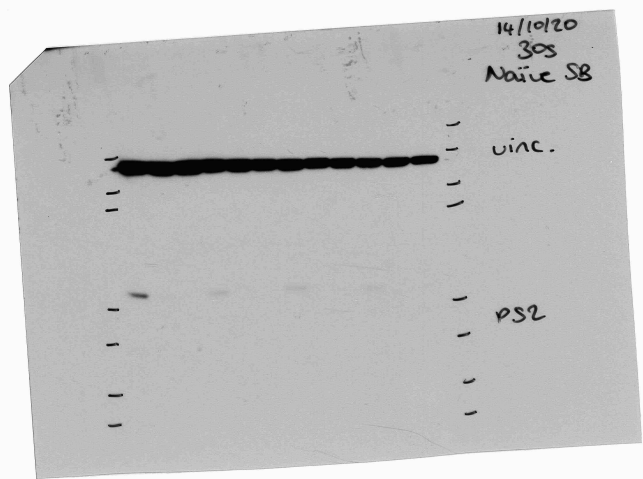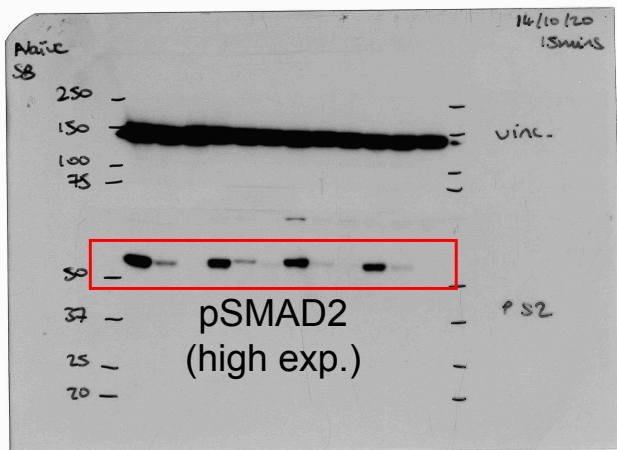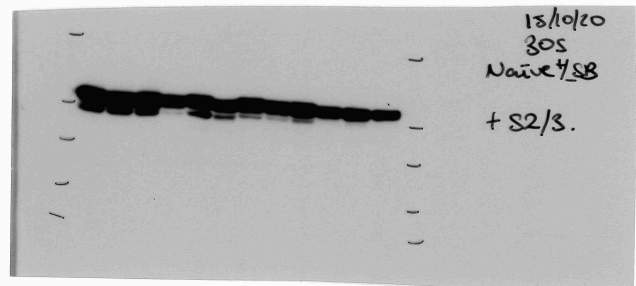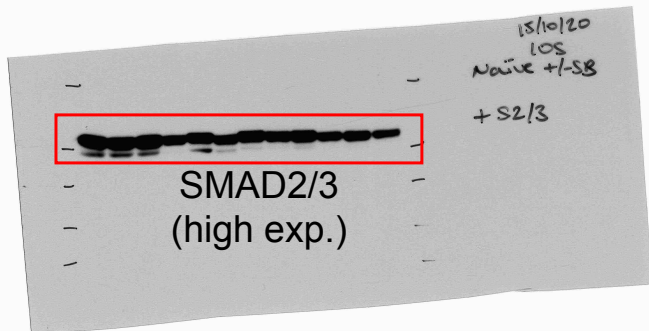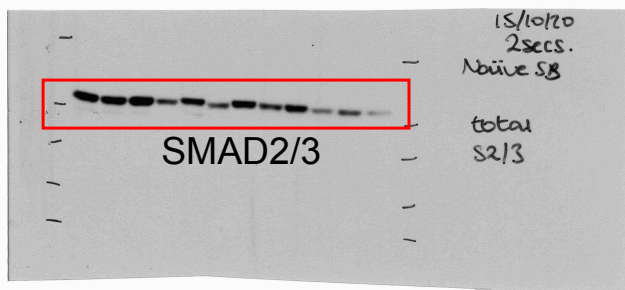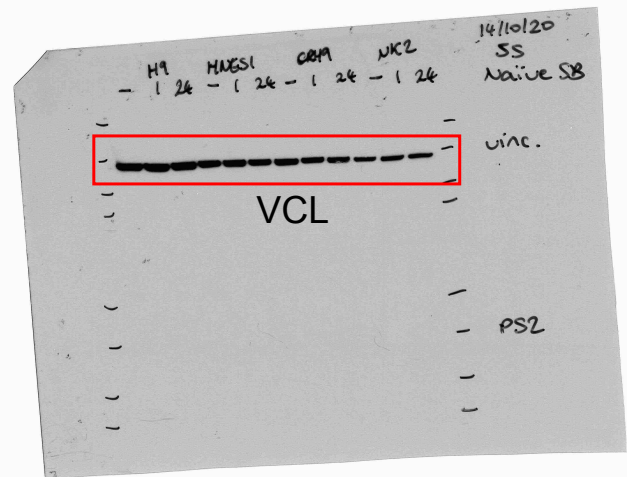

Supplement: Figure 1—source data 1. — Blots show SMAD2 phosphorylation signal and total SMAD2/3 in normal conditions (-), and following 1 hr and 24 hr of SB-431542 supplementation to their culture media. Two separate exposures are shown, Vinculin (VCL) used as loading control. (a) Original files of the full raw unedited blots. (b) Figures with the uncropped blots with the bands reported in the main figures highlighted in red and labelled accordingly. [file elife-67259-fig1-data1.pdf]

Figure 3 - Figure supplement 1 - Source data 1

a

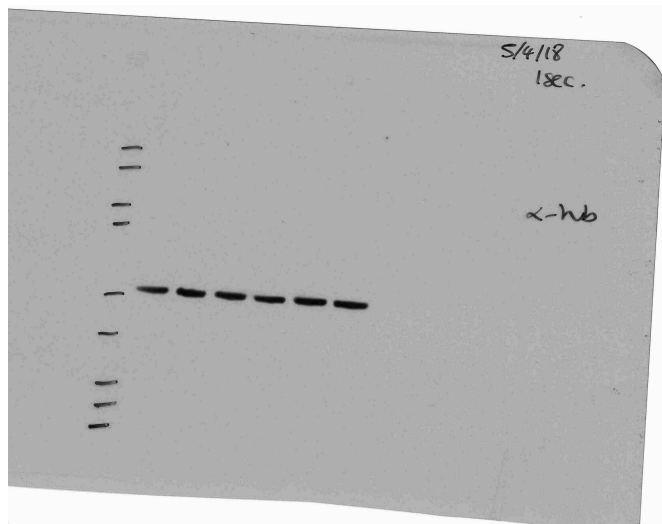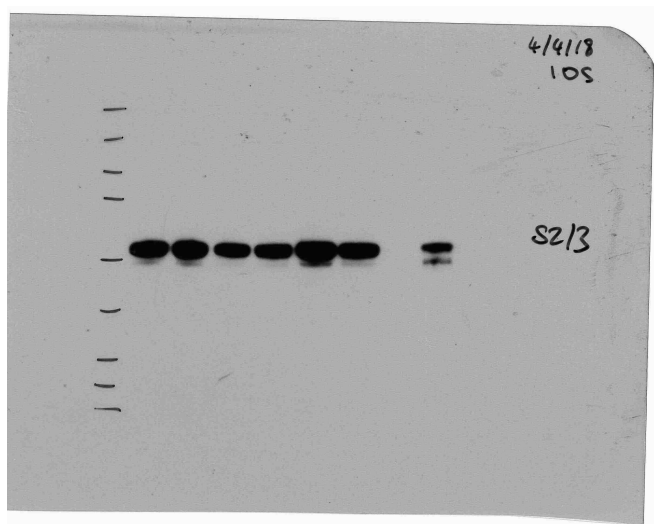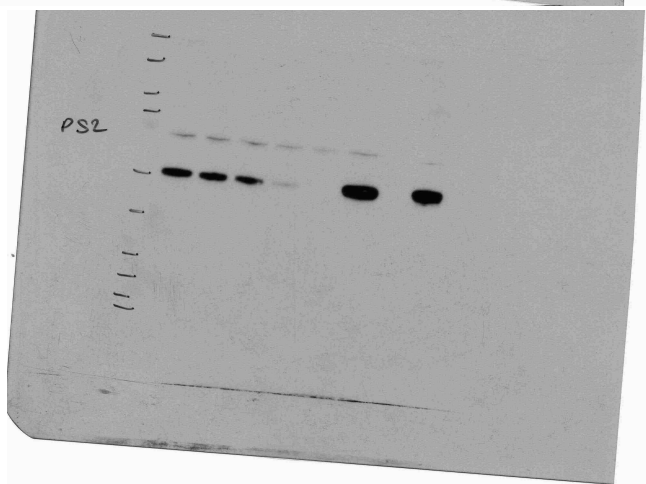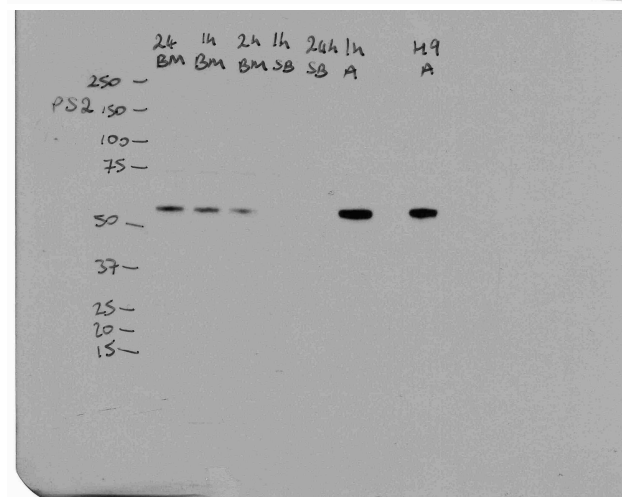

**b**

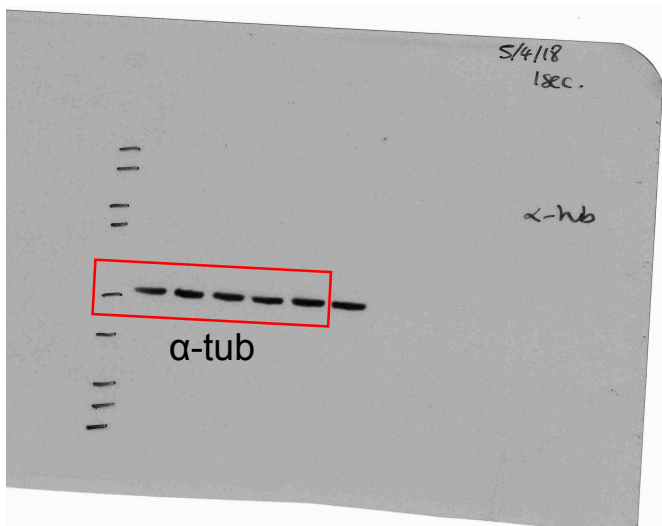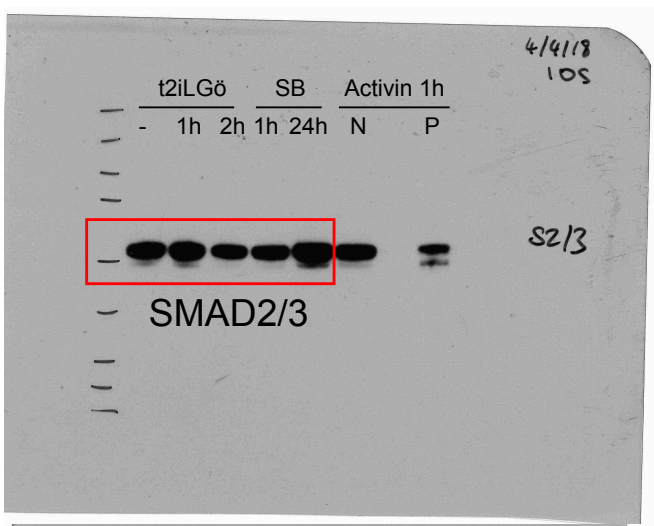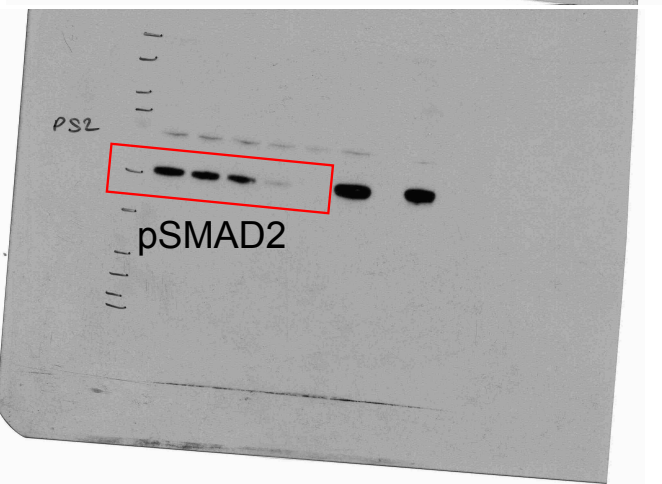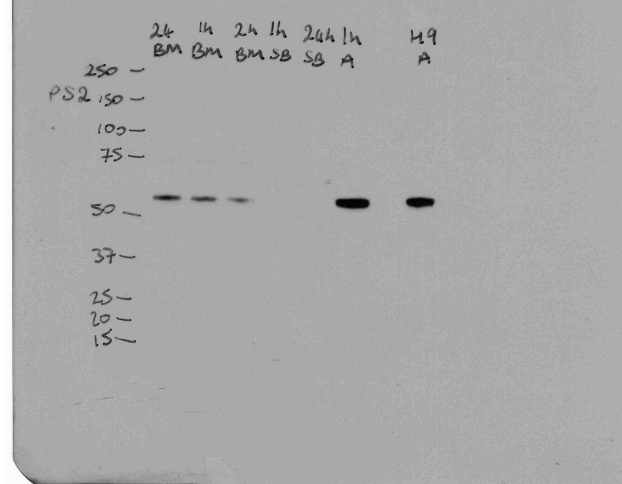

Supplement: Figure 3—figure supplement 1—source data 1. — Alpha-tubulin (α-tub) used as loading control. Additional lanes (not shown in the main figure for clarity) report as positive control naïve (N) and primed (P) hPSC treated for 1 hr with Activin. (a) original files of the full raw unedited blots. (b) figures with the uncropped blots with the bands reported in the main figures highlighted in red and labelled accordingly. [file elife-67259-fig3-figsupp1-data1.pdf]
