# Supplementary material for: TGFβ signalling is required to maintain pluripotency of human naïve pluripotent stem cells
Source: eLife. 2021 Aug 31;10:e67259. doi: 10.7554/eLife.67259 (PMC8410071; doi:10.7554/eLife.67259)
Supplement: Transparent reporting form [file elife-67259-transrepform.docx]

***eLife’s* transparent reporting form**

We encourage authors to provide detailed information *within their submission* to facilitate the interpretation and replication of experiments. Authors can upload supporting documentation to indicate the use of appropriate reporting guidelines for health-related research (see [EQUATOR Network](http://www.equator-network.org/%20)), life science research (see the [BioSharing Information Resource](https://biosharing.org/)), or the [ARRIVE guidelines](http://www.plosbiology.org/article/info:doi/10.1371/journal.pbio.1000412) for reporting work involving animal research. Where applicable, authors should refer to any relevant reporting standards documents in this form.

If you have any questions, please consult our Journal Policies and/or contact us: [editorial@elifesciences.org](mailto:editorial@elifesciences.org).

**Sample-size estimation**

- You should state whether an appropriate sample size was computed when the study was being designed
- You should state the statistical method of sample size computation and any required assumptions
- If no explicit power analysis was used, you should describe how you decided what sample (replicate) size (number) to use

Please outline where this information can be found within the submission (e.g., sections or figure legends), or explain why this information doesn’t apply to your submission:

No sample size calculations were performed. The number of replicates were estimated based on our experience with these techniques and standard practices in the field.

Sample size is stated in the figure legends and/or in the methods section for each experiment.

Sample size: for qPCR data, three biological replicates for each experiment were performed, unless otherwise stated in the figure legends; for ChIP-seq data, two biological replicates with corresponding input were performed, as per ENCODE guidelines (https://www.encodeproject.org/chip-seq/transcription_factor/).

**Replicates**

- You should report how often each experiment was performed
- You should include a definition of biological versus technical replication
- The data obtained should be provided and sufficient information should be provided to indicate the number of independent biological and/or technical replicates
- If you encountered any outliers, you should describe how these were handled
- Criteria for exclusion/inclusion of data should be clearly stated
- High-throughput sequence data should be uploaded before submission, with a private link for reviewers provided (these are available from both GEO and ArrayExpress)

Please outline where this information can be found within the submission (e.g., sections or figure legends), or explain why this information doesn’t apply to your submission:

Experiments in biological replicate (RT-qPCRs n=3, ChIP-seq n=2) were performed independently on cell lines from different passages. High-throughput sequencing was carried out in the same sequencing run. Samples from different cell lines for western blot have been collected on different days, and run simultaneously on the same gel. The replicates for the H9 NK2 line were collected on different days and were run independently.

Biological replicates were defined as separate experiments using the same line from different passages performed at different times, or experiments run simultaneously on three different cell lines, as specified in the methods. Technical replicates in RT-qPCR experiments are defined as parallel wells of the same cell line in the same experiment.

The number of replicates is specified in each figure legend and relevant methods section.

We did not exclude outliers from any analysis.

High-throughput sequencing data is publicly available.

**Statistical reporting**

- Statistical analysis methods should be described and justified
- Raw data should be presented in figures whenever informative to do so (typically when N per group is less than 10)
- For each experiment, you should identify the statistical tests used, exact values of N, definitions of center, methods of multiple test correction, and dispersion and precision measures (e.g., mean, median, SD, SEM, confidence intervals; and, for the major substantive results, a measure of effect size (e.g., Pearson's r, Cohen's d)
- Report exact p-values wherever possible alongside the summary statistics and 95% confidence intervals. These should be reported for all key questions and not only when the p-value is less than 0.05.

Please outline where this information can be found within the submission (e.g., sections or figure legends), or explain why this information doesn’t apply to your submission:

Statistical analysis. For RT-qPCRs, ANOVA with Tukey's or Šídák's multiple comparisons test was performed in order to allow multiple comparisons between means. Data show the mean ± SD of three biological replicates (unless specified in figure legend). For scRNA-seq differential expression analysis, Wilcoxon Rank Sum test was used, with FDR < 0.05 and Benjamini-Hochberg correction for the single-cell heatmaps. For overlap between the ChIP-seq and scRNA-seq datasets, Chi-square test with Yates continuity and Bonferroni multiple testing correction were used.

Single data points are shown when n is less than 10.

P-values are included in the graphs when relevant.

Statistical analysis methods and n are specified in the figure legends for each graph and/or in methods section. Numerical data are reported in the source data files.

(For large datasets, or papers with a very large number of statistical tests, you may upload a single table file with tests, Ns, etc., with reference to sections in the manuscript.)

**Group allocation**

- Indicate how samples were allocated into experimental groups (in the case of clinical studies, please specify allocation to treatment method); if randomization was used, please also state if restricted randomization was applied
- Indicate if masking was used during group allocation, data collection and/or data analysis

Please outline where this information can be found within the submission (e.g., sections or figure legends), or explain why this information doesn’t apply to your submission:

Individual tissue culture wells were randomly allocated to control or inhibitor treatment groups at the start of each experiment. No masking was used in the experiments.

**Additional data files (“source data”)**

- We encourage you to upload relevant additional data files, such as numerical data that are represented as a graph in a figure, or as a summary table
- Where provided, these should be in the most useful format, and they can be uploaded as “Source data” files linked to a main figure or table
- Include model definition files including the full list of parameters used
- Include code used for data analysis (e.g., R, MatLab)
- Avoid stating that data files are “available upon request”

Please indicate the figures or tables for which source data files have been provided:

Numerical data and Western blot images are reported in the source data files.

Sequencing data are publicly available.
